# Giant Aneurysm of Coronary Artery Fistula Successfully Treated by Surgical Repair

**DOI:** 10.7759/cureus.7430

**Published:** 2020-03-27

**Authors:** Yuko Harada, Atsuo Mori, Mitsuharu Mori, Tatsuji Yoshimoto

**Affiliations:** 1 Internal Medicine, Yamato Tokushukai Hospital, Yamato, JPN; 2 Internal Medicine, Harada Naika Clinic, Kawasaki, JPN; 3 Cardiovascular Surgery, Kawasaki Municipal Hospital, Kawasaki, JPN; 4 Cardiovascular Surgery, Saiseikai Yokohamashi Tobu Hospital, Yokohama, JPN; 5 Cardiology, Kawasaki Municipal Ida Hospital, Kawasaki, JPN

**Keywords:** coronary artery fistula, giant aneurysm, coronary artery bypass graft surgery

## Abstract

An asymptomatic 65-year-old woman was identified with an oversized round-shaped hypoechoic lesion (62 mm in diameter) between right and left atria by echocardiogram. A contrast-enhanced 320-slice multidetector computed tomography demonstrated a giant aneurysmatic fistula branched from the left main coronary trunk towards right atrium. The patient underwent an elective surgical repair. The aneurysm was resected, followed by coronary artery bypass graft surgery using bilateral internal thoracic arteries. The surgery was successful, and the patient enjoys normal life without any symptoms for 15 months.

## Introduction

Coronary artery fistula (CAF) is a relatively rare anatomic abnormality of the coronary arteries that afflicts 0.002% of the general population and represents 14% of all the anomalies of coronary arteries [1]. CAF is sometimes presented with aneurysms without any symptoms. A giant aneurysmal formation in CAF is extremely rare, and presents high risk of rupture. Treatment strategy differs by patient according to size and location of anomalous vessel. We encountered the present case of giant aneurysm formed in CAF, and the patient underwent elective surgery with success.

## Case presentation

**Figure 1:**
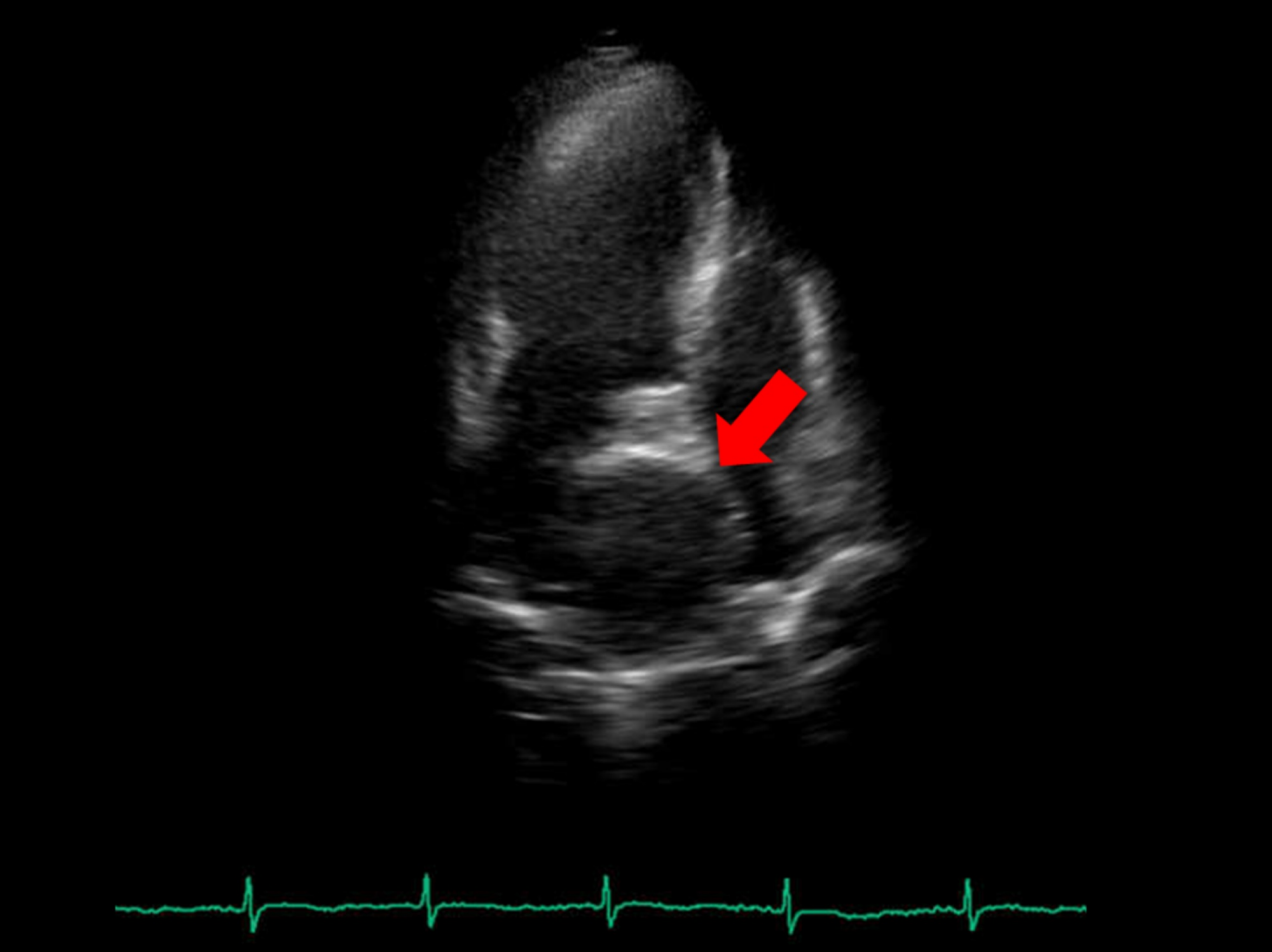
Echocardiogram before surgery. Transthoracic echocardiogram showing a round-shaped hypoechoic lesion (red arrow) of 62 mm diameter between right and left atria.

**Figure 2:**
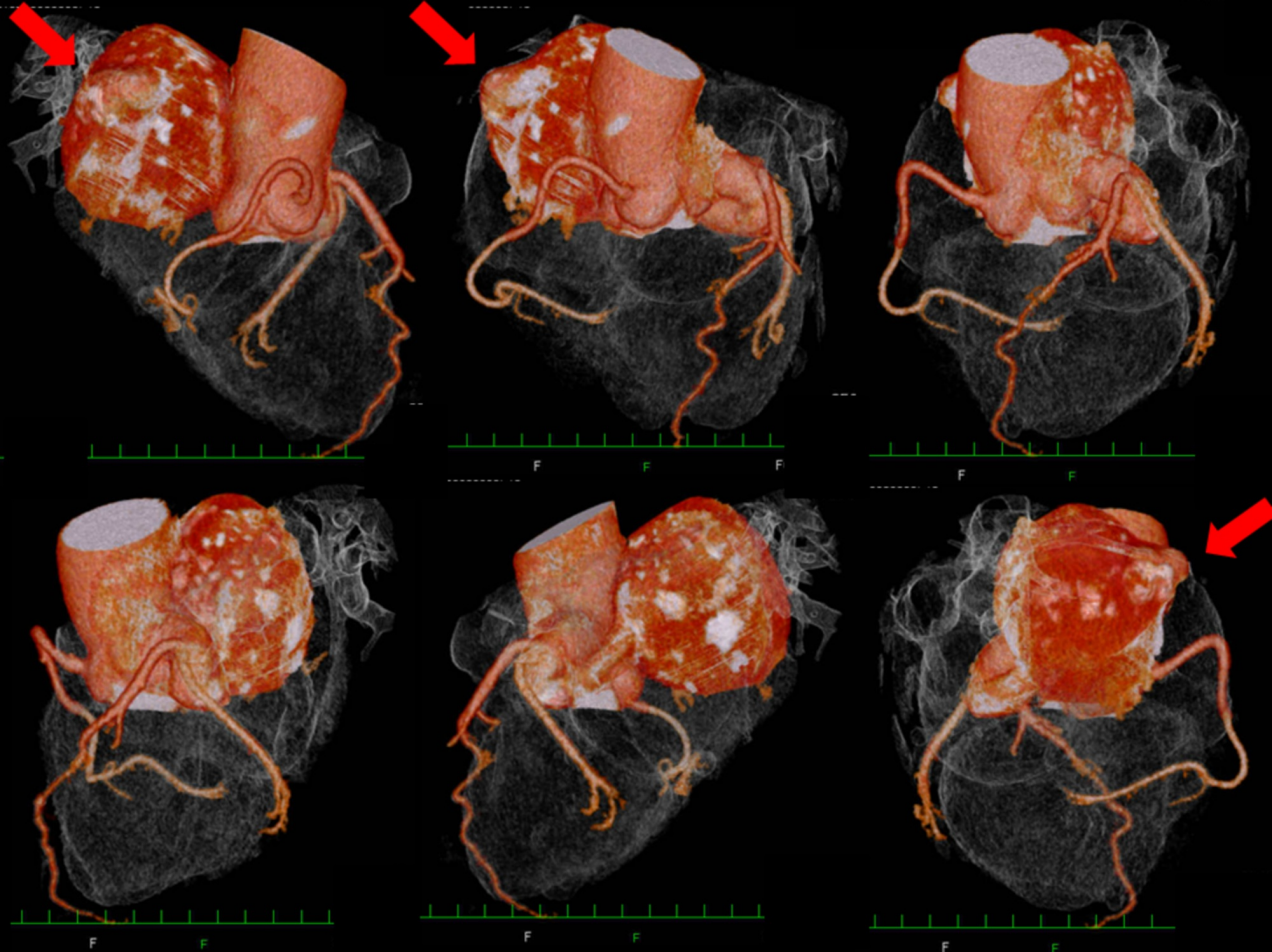
MDCT before surgery. MDCT rotational view (from top left to bottom right). Enlarged LMT branches LAD and LCX, then runs dorsally towards RA forming giant aneurysm (bottom, middle). A bulging limp (red arrow) in the aneurysm turned out to be the opening to RA. LAD, left anterior descending artery; LCX, left circumflex coronary artery; LMT, left main coronary trunk; MDCT, multidimensional computed tomography; RA, right atrium.

A 65-year-old woman visited our hospital for health checkup. She presented no symptoms, no chest pain, and no dyspnea on exertion. Electrocardiogram was normal; however, chest X-ray revealed an enlarged heart, without any sign of atherosclerosis. Transthoracic echocardiography revealed a round-shaped hypoechoic lesion of 62 mm in diameter between right and left atria (Figure 1). Multidimensional computed tomography (MDCT) revealed lesion as a giant aneurysm of 58 mm in diameter with mural thrombus formed in the dilated winding vessel arising from left main coronary trunk (LMT) (Figure 2). The entire LMT indicated dilation. Both left anterior descending artery (LAD) and left circumflex coronary artery (LCX) displayed neither stenosis nor dilation. The right coronary artery was shown intact.

Coronary angiography confirmed the findings of MDCT, but the outflow of the aneurysm was not identified.

Considering the size of aneurysm, aneurysmal resection by open heart surgery to prevent rupture was recommended. The patient consented to undergo surgery.

**Figure 3:**
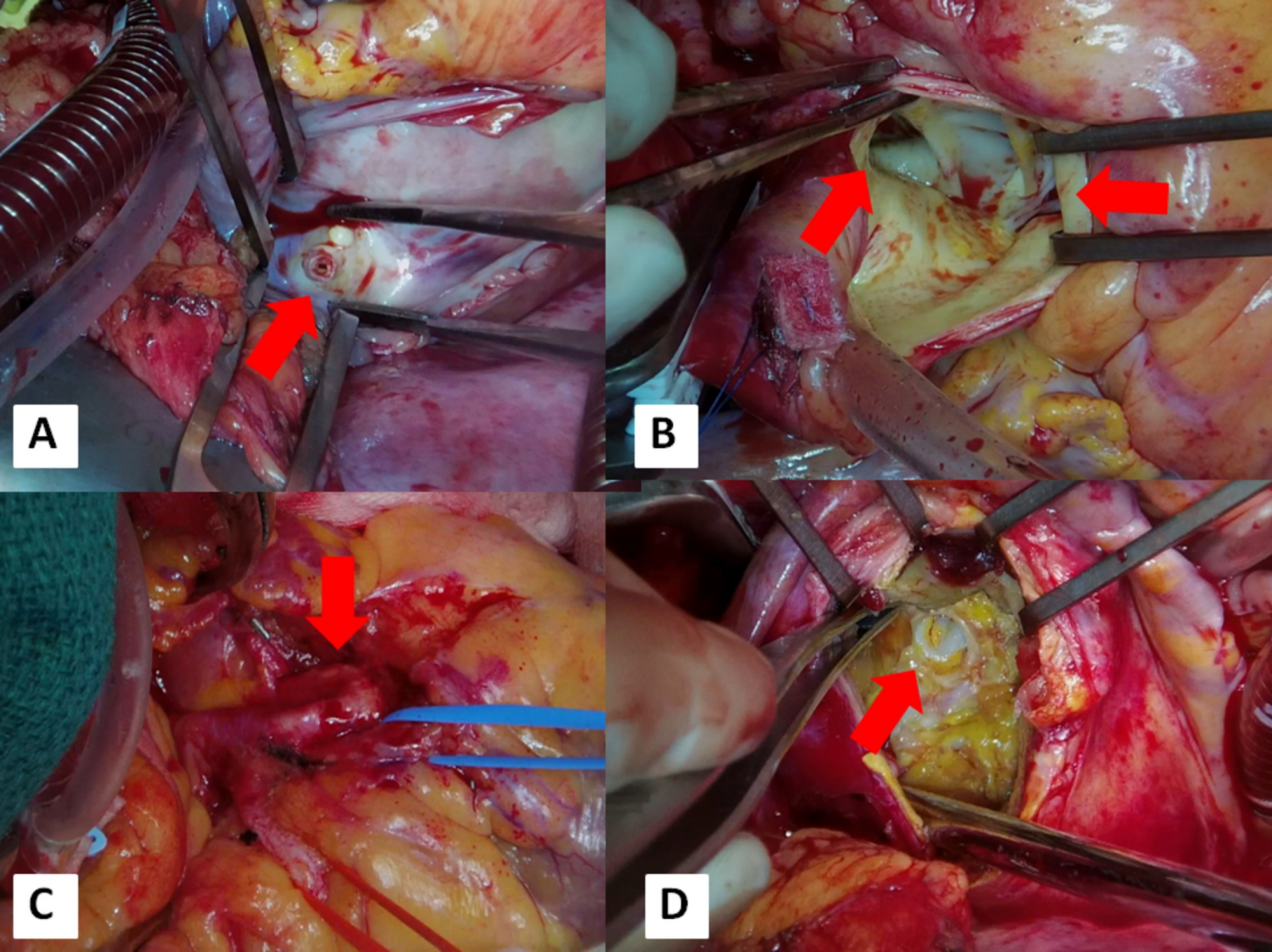
Surgical findings. Upper left quadrant image A: inside RA. The inlet from the fistula is observed (arrow). The inlet was ligated. Upper right quadrant image B: inside the aortic root observing aortic valve (right arrow) and the opening of LMT (left arrow). Opening of LMT was ligated. Lower left quadrant image C: enlarged LMT branching LAD (red tape) and LCX (blue tape). Bypass surgery was operated. Lower right quadrant image D: inside aneurysm. The inlet from LMT is observed (arrow). LAD, left anterior descending artery; LCX, left circumflex coronary artery; LMT, left main coronary trunk; RA, right atrium.

Intraoperative inspection revealed outflow of aneurysm drainage into right atrium (RA) (Figure 3A). No branch vessel of fistula feeding the myocardium was identified. No branch vessel of aneurysm was identified. Both ends of the fistula vessel were ligated: the draining end at RA and the ostium of LMT (Figure 3B). Then bypass surgeries of the left internal thoracic artery to LAD and the right internal thoracic artery to LCX were performed (Figure 3C). Incision into aneurysm was made and mural thrombus was removed, thereby revealing inflow from LMT (Figure 3D).

**Figure 4:**
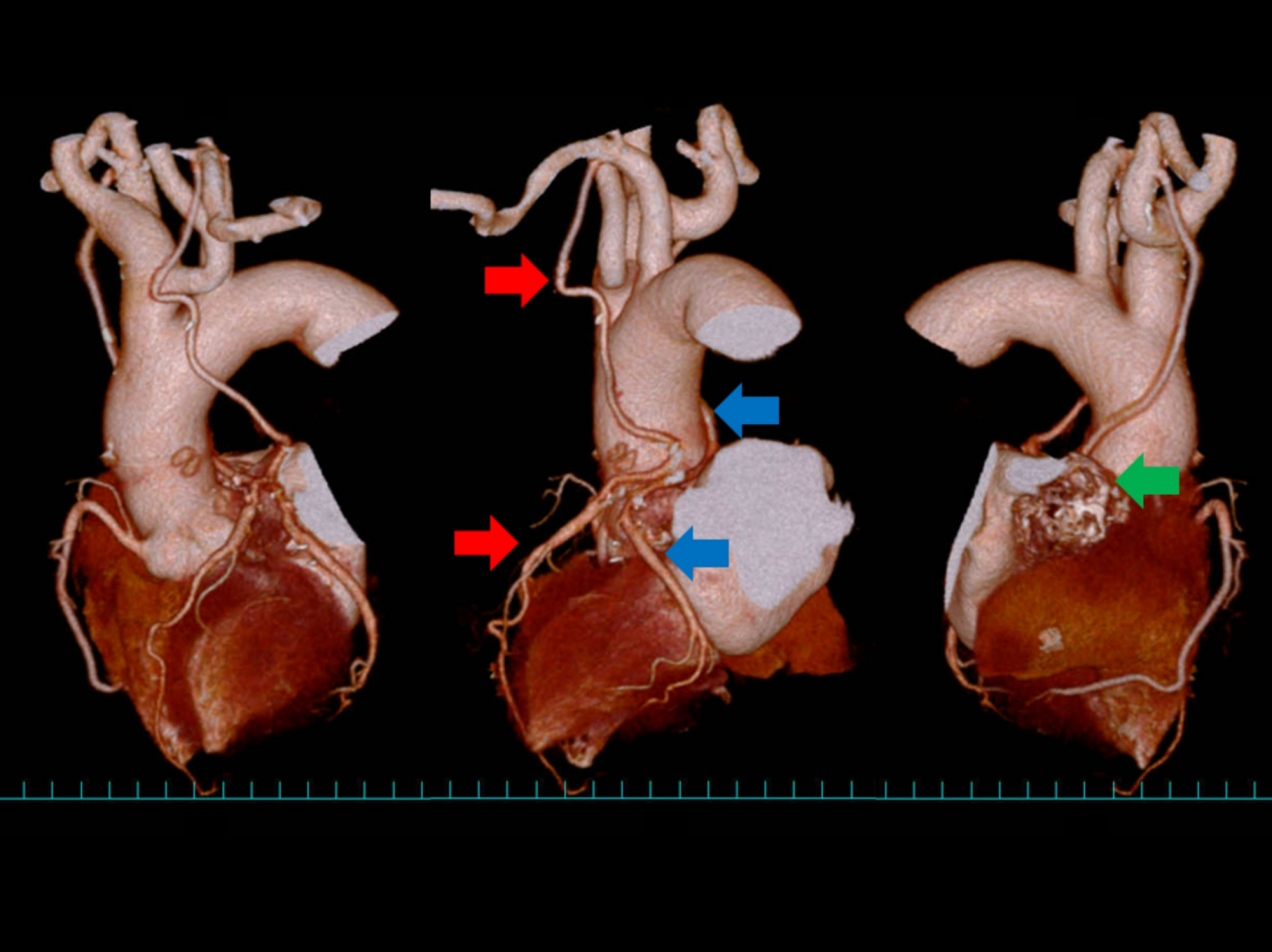
One year after surgery. MDCT rotational view (from left to right). Both bypass vessels were intact. Red arrow: LITA-LAD bypass. Blue arrow: RITA-LCX bypass. Green arrow: shrunk aneurysm. LAD, left anterior descending artery; LCX, left circumflex coronary artery; LITA, left internal thoracic artery; MDCT, multidimensional computed tomography; RITA, right internal thoracic artery.

After surgery, the patient had paroxysmal atrial fibrillation which was controlled by amiodarone. The patient has been in good health without any cardiovascular events for one year thereafter. Contrast CT one year post surgery showed that coronary artery aneurysm had disappeared and bypass vessels were intact (Figure 4).

## Discussion

CAF is a relatively rare congenital anomaly, but has been detected more frequently due to coronary angiography being performed more frequently over the last few decades. Approximately 20% of CAF are accompanied by coronary artery aneurysm [2]. Our case was very unusual because the entire CAF was extremely dilated with its end bulged into giant aneurysm. The cause of aneurysm was unknown, but a childhood incidence of Kawasaki disease was suspected because the patient was otherwise healthy. Alternative hypothesis was that the vessel wall of CAF lacked elastic fiber, leading to aneurysm to be formed in the weakened or stressed vessel wall.

CAF from LMT to RA, such as in this case, is rare among CAF patients. A review article revealed that unilateral fistulas originated from LMT comprised 7% of 243 cases with CAF [3]. Among fistulas originated from LMT, 29% terminated in the RA or coronary sinus. Therefore, CAF from LMT to RA calculates to 2.0% of all CAF patients.

CAF without symptoms are usually not considered an indication for surgical treatment. However, in recent years there have been several case reports of ruptured aneurysm in CAF that underwent surgery [4-7]. Rittenhouse et al. advocated that a large shunt flow (average Qp/Qs = 2.0) and symptoms of heart failure are justified indications for surgery, but left-to-right shunt flow is usually low (average Qp/Qs = 1.5) and coronary artery steal syndrome is rare [8]. The risk factors for rupture of congenital aneurysmal fistula are female gender, saccular aneurysm, Asian ethnicity, origin of aneurysmal fistulas from left coronary artery, and hypertension [9]. Coronary artery aneurysms larger than 30 mm in diameter are considered to be at increased risk of rupture [10]. Thus, our reported case was highly likely at risk of rupture due to the size, female gender, Asian ethnicity, and its origin of LMT.

Lowe et al. stated that surgical management is appropriate in symptomatic patients who displayed evidence of emboli from the aneurysm to the distal coronary bed leading to myocardial ischemia, and also in cases of aneurysmal enlargement as documented by serial angiographic measurement [11]. They also stated that patients not managed surgically must be monitored very closely (every three months) and treated with antiplatelet and anticoagulation therapy to prevent thrombus formation within the aneurysm. Our patient was asymptomatic, displaying neither chest pain nor dyspnea. However, considering the extraordinary size of aneurysm, surgery was considered to be optimal for this reported case in order to prevent future catastrophic cardiovascular events. The surgery revealed massive mural thrombus in the aneurysm which was almost occluded; thus, the successful surgery saved the patient’s life. Even if a patient of CAF with aneurysm is asymptomatic, detailed evaluation by MDCT is essential, and surgical treatment should be considered.

## Conclusions

A giant aneurysm in the CAF was detected during standard health checkup. MDCT successfully revealed the anomaly and its structure. As a result, open-heart surgery confirmed the findings of MDCT and saved the patient's life.
